# Analysis of the efficacy of transesophageal atrial pacing in the treatment of supraventricular tachycardia in newborns

**DOI:** 10.3389/fcvm.2026.1866714

**Published:** 2026-08-03

**Authors:** Meng Xu, Tingting Xiao, Chongbing Yan, Yan Han, Zhen Yan, Cuilan Hou, Xiaopei Zhao, Li Zhang

**Affiliations:** 1 Department of Cardiology, Shanghai Children's Hospital, School of Medicine, Shanghai Jiao Tong University, Shanghai, China; 2 Department of Neonatology, Shanghai Children's Hospital, School of Medicine, Shanghai Jiao Tong University, Shanghai, China

**Keywords:** treatment, newborn, supraventricular tachycardia, transesophageal atrial pacing, transesophageal electrocardiography

## Abstract

**Objective:**

To describe and explore the therapeutic value of transesophageal atrial pacing (TEAP) for supraventricular tachycardia in newborns.

**Methods:**

Clinical data from 22 cases (15 males, 7 females) at Shanghai Children's Hospital (2015–2025) were retrospectively analyzed to evaluate the efficacy of TEAP.

**Results:**

Surface ECGs demonstrated paroxysmal SVT and type I atrial flutter in 11 cases respectively, with 8 patients having heart failure. After inserting the esophageal electrode catheter, transesophageal electrocardiography confirmed Atrioventricular Reentrant Tachycardia (AVRT) in 9 of 11 SVT cases; TEAP successfully terminated tachycardia in 7 (77.8% success). In a case, pulse stimulation had not yet been administered, a declining trend in blood pressure was observed, which necessitated electrical cardioversion. Another with short paroxysms post-TEAP was maintained on oral medication. Two cases of atrial tachycardia confirmed by transesophageal electrocardiogram did not respond to TEAP. Among 11 type I atrial flutter cases, TEAP restored sinus rhythm in 9 (81.8% success); one had intermittent short paroxysms and one showed reduced atrioventricular conduction, both switched to oral drugs.

**Conclusion:**

TEAP is a relatively safe and effective acute treatment for reentrant SVT (AVRT, AFL) when medication cannot promptly and effectively terminate tachycardia, particularly for patients with heart failure.

## Introduction

Supraventricular tachycardia (SVT) is the most common sustained arrhythmia in newborns (1). Persistent tachycardia can lead to heart failure, cardiogenic shock, and even death. Currently, medication is the primary method for treating tachyarrhythmias in newborns. However, pharmacological management presents unique challenges due to significant developmental differences in pharmacokinetics between newborns and older children, as well as factors such as low body weight and small organ size.

TEAP is a minimally invasive electrophysiological technique widely used to assess the induction and termination of tachycardia (2). This technique leverages the close anatomical proximity between the esophagus and the left atrium to achieve indirect atrial pacing through electrical pulse delivery. This technique is especially suitable for the evaluation and management of supraventricular tachycardia in newborns and infants (3). However, reports on TEAP for treating SVT in newborns remain scarce in recent years. This study summarizes the clinical data of 22 cases and analyzes them. This study is descriptive and exploratory given the small sample size and heterogeneity of arrhythmia mechanisms.

## Methods

### Study objects

This study retrospectively included 22 newborns with SVT admitted to Shanghai Children's Hospital from January 2015 to December 2025, comprising 15 males and 7 females. Inclusion criteria: (1) Age ≤ 28 days; (2) Surface electrocardiogram indicating SVT, originating from above the His bundle, including paroxysmal supraventricular tachycardia (PSVT), atrial tachycardia (AT), and atrial flutter (AFL) (4, 5); (3) Defined as SVT persisting despite the administration of at least 1 class of antiarrhythmic agents at appropriate doses and durations (e.g., each drug trial lasting for at least 2 h). Exclusion criteria: (1) Intermittent, short-duration paroxysms of SVT at admission; (2) Tachycardia responsive to vagal maneuvers or medication; (3) Chaotic atrial tachycardia, atypical atrial flutter, and atrial fibrillation.

### Research methods

Collect demographic characteristics, clinical manifestations, results of laboratory and auxiliary examinations (including surface electrocardiogram, echocardiogram, and blood biomarkers), TEAP, and related pharmacological interventions for the newborns.

#### Antiarrhythmic drug treatment

(1) For SVT not terminated by vagal maneuvers (e.g., applying a cold water towel to the face for 10–15 s), rapid intravenous injection of ATP is the preferred treatment. If this proves ineffective, infants with normal cardiac function should receive propafenone, whereas those with heart failure should be administered amiodarone. (2) For patients with atrial flutter and normal cardiac function, digoxin or rhythm-control agents are administered, often in combination with oral beta-blockers. In contrast, patients with heart failure are treated with digoxin or amiodarone.

#### TEAP

TEAP is performed promptly if antiarrhythmic drug treatment fails to effectively terminate tachycardia, especially in patients with heart failure. All patients' families signed informed consent forms, and fasting was required for 2 h before TEAP. A DF-5A cardiac electrophysiological stimulator (Suzhou Oriental Electronic Instrument Factory) was used, and the depth of the catheter was marked from the nasal tip to the xiphoid process before insertion. A 7F quadripolar esophageal electrode catheter was inserted orally (although in some newborns weighing >3 kg, the catheter was inserted nasally). The catheter position was adjusted to maximize the amplitude of biphasic or multiphasic *P* waves on the esophageal electrocardiogram, ensuring ideal positioning and fixation at an insertion depth of 13–20 cm. The output voltage was gradually increased until atrial pacing was achieved (pacing voltage: 5–12 V; pulse width: 10 ms), ensuring effective atrial capture throughout the procedure. Restoration methods: (1) The rapid suppression method was employed, utilizing an S1S1 frequency 20%–30% higher than the atrial rate during tachycardia, with delivery of 3–10 stimuli. If tachycardia is not terminated, intervals of 30–60 s are taken, gradually increasing the S1S1 stimulation frequency to 20–50 times/min until tachycardia is terminated (maximum stimulation frequency for PSVT 400 bpm, for AFL 600 bpm); (2) Burst stimulation. Throughout the procedure, relevant emergency medications and a cardiac defibrillator were prepared to manage potential complications. TEAP success was defined as acute termination of SVT (conversion to sinus rhythm) without early recurrence (within 24 h).

### Statistical methods

Statistical analyses were performed using SPSS version 29.0. Continuous variables with a normal distribution are presented as mean ± standard deviation. While those with a non-normal distribution are reported as median and interquartile range (P25–P75). Count data are expressed as case numbers (percentage), and inter-group comparisons are conducted using the *χ*^2^ test. *P* < 0.05 was considered statistically significant.

## Results

### Clinical characteristics

Among the 22 newborns with SVT and drug therapy failed to terminate in time, 15 were male and 7 were female; gestational age ranged from 32 + 4 to 40 + 5 weeks (mean 36.76 ± 2.82 weeks), age at first diagnosis from 1 to 28 days (mean 11.48 ± 10.91 days), and weight from 2,170 to 5,320 g (mean 3,373.18 ± 847.76 g). The ventricular rate during tachycardia ranged from 175 to 300 beats/min (mean 224.09 ± 40.99 beats/min). There were 14 term infants and 8 late preterm infants. Prenatal monitoring revealed fetal tachycardia or fetal distress in 13 cases. Associated underlying conditions included congenital heart disease, perinatal asphyxia, pathological jaundice, and infections. Eight patients presented with heart failure, and no new cases developed during treatment. After inserting the esophageal electrode catheter, in a case pulse stimulation had not yet been administered, a declining trend in blood pressure was observed, which necessitated electrical cardioversion (see Table 1). The main symptoms of SVT in newborns included lethargy, crying, pallor, cyanosis, dyspnea, or poor feeding, which were also found during physical examination.

**Table 1:** Comparison of clinical data on neonatal AVRT, AT, and AFL cases.

Clinical characteristics	AVRT	AT	AFL
Gender (Male/Female, *n*)	5/4	1/1	9/2
Gestational Week (Week)	36.89 ± 3.11	36.35 ± 3.09	36.79 ± 2.81
Age (day)	13.57 ± 11.89	15.02 ± 21.21	11.27 ± 10.91
Weight (g)	3,481.11 ± 731.46	3,300 ± 1,484.92	3,298.18 ± 914.55
Late preterm infants	2	2	4
Fetal Tachycardia/Fetal Distress	3	2	8
Atrial Rate (beats per minute)	261.22 ± 32.85	199.50 ± 14.85	400.40 ± 47.49
Ventricular Rate (beats per minute)	261.22 ± 32.85	199.50 ± 14.85	198.18 ± 23.17
CK-MB (U/L)	220.56 ± 279.13	229.50 ± 268.00	103.10 ± 77.00
cTNI (ng/mL)	0.11 ± 0.09	0.05 ± 0.01	0.07 ± 0.04
NT-BNP (pg/mL)	1,930.75 ± 1,183.60	1,641.00 ± 1,213.40	7,339.56 ± 8,311.81
Comorbid underlying diseases:			
Congenital Heart Disease	5	2	1
Other (asphyxia, jaundice, infection, etc.)	6	1	9
Cardiac functional insufficiency	5 (1 case with cardiogenic shock)	1	2
TEAP cardioversion (success/failure, *n*)	7/2	0/2	9/2
Electrical conversion	1	0	0

### TEAP treatment and outcomes

**Figure 1:**
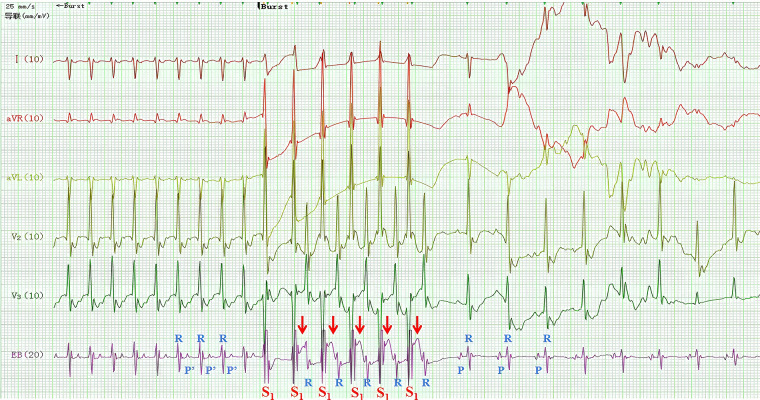
Surface ECG and esophageal ECG synchronous recording of PSVT: lead EB (20 mm/mV), when tachycardia occurs RR interval 256 ms (234 bpm), RP' 104 ms, P'R 156 ms, suggestive of AVRT; S1S1 interval 333 ms (180 bpm), 6 burst stimuli, clearly visible atrial capture by the 2nd to 6th S1 stimuli (indicated by red arrow), followed by ventricular conduction, forming a trailing phenomenon, termination of tachycardia, and restoration of sinus rhythm.

**Figure 2:**
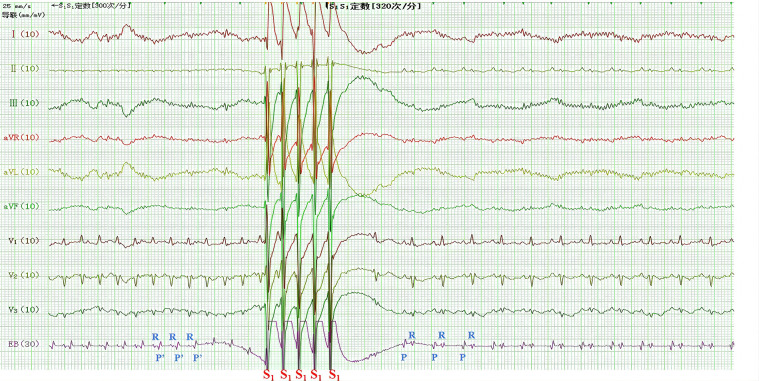
Surface electrocardiogram: PSVT, low voltage. Synchronized recording of lead E (30 mm/mV), when tachycardia occurs P' wave amplitude > R wave amplitude, RR interval 216 ms (277 bpm), RP' 80 ms, P'R 136 ms; S1S1 interval 188 ms (320 bpm), S1S1 stimulation *5 times, termination of AVRT, restoration of sinus rhythm.

Of the eleven newborns diagnosed with PSVT via surface electrocardiogram, nine were confirmed to have AVRT by transesophageal electrocardiography (see Table 1); these cases exhibited an RP' interval >70 ms and an RP' interval shorter than the P'R interval, with no instances of Atrioventricular Nodal Reentrant Tachycardia (AVNRT) observed. Sinus rhythm was successfully restored in seven newborns with AVRT using TEAP, yielding a success rate of 77.8% (7/9); the rapid suppression method terminated tachycardia in six cases, while burst pacing terminated it in one case (see Figures 1, 2). In one case of recurrent persistent tachycardia, an esophageal electrode catheter was maintained for 24 h; during this period, three pacing sessions (consisting of 8, 6, and 2 attempts, respectively) were performed, each successfully terminating the tachycardia and resulting in the complete restoration of sinus rhythm after 24 h. A patient with ventricular preexcitation-induced supraventricular tachycardia experienced two recurrence episodes after discharge and was treated with oral propafenone for maintenance therapy.

**Figure 3:**
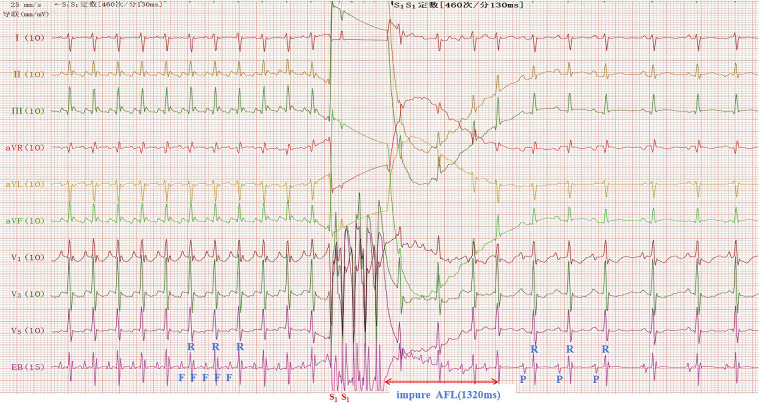
Surface ECG and esophageal ECG were recorded synchronously. The ECG parameters were as follows: EB lead (15 mm/mV), FF interval 137 ms (436 bpm), RR interval 274 ms (218 bpm), atrioventricular conduction ratio 2:1, S1S1 interval 130 ms (460 bpm). After 5 S1S1 stimuli, the rhythm transitioned to impure atrial fibrillation (AFL). Sinus rhythm was restored after 1,320 ms.

**Figure 4:**
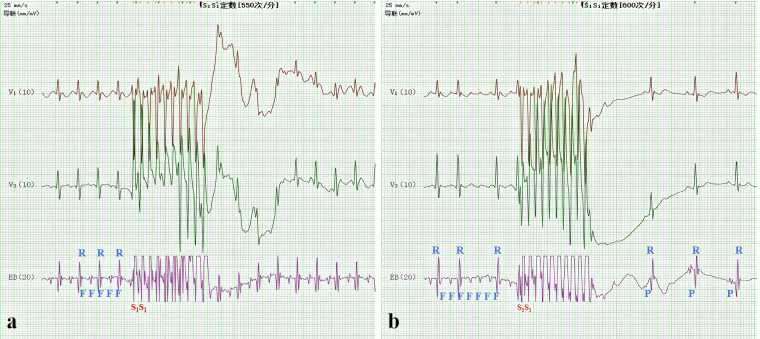
**(a)** AFL with a 2:1 atrioventricular conduction ratio, AFL-FF interval of 140 ms (428 bpm), RR interval of 280 ms (214 bpm), S1S1 interval of 109 ms (550 bpm). Ten consecutive S1S1 stimulations at this interval failed to terminate the AFL episode. **(b)** In the same patient during TEAP treatment, AFL converted to 2∼4:1 conduction. Increasing the stimulation frequency to an S1S1 interval of 100 ms (600 bpm), ten consecutive S1S1 stimulations resulted in an 880 ms long RR interval, terminating the AFL and restoring sinus rhythm.

TEAP restoration failed in two newborns with AVRT. The first, who had congenital heart disease complicated by heart failure, pulse stimulation had not yet been administered, a declining trend in blood pressure was observed; this patient required conversion to electrical cardioversion (1 J·kg⁻^1^ administered three times) to completely terminate the episode. The second patient presented with short paroxysms of tachycardia following TEAP treatment and was prescribed oral rhythm-control medication. Two cases diagnosed as PSVT by surface electrocardiogram were confirmed as atrial tachycardia by transesophageal electrocardiography (see Table 1), with esophageal leads showing P'R < RP', P'R < 1/2RR, and TEAP was unsuccessful; consequently, both patients were switched to oral digoxin plus beta-blocker therapy. Surface electrocardiogram diagnosed 11 cases of type I atrial flutter (10 cases with AV conduction ratio of 2:1, 1 case with AV conduction ratio of 2:1∼3:1) (see Table 1). TEAP terminated atrial flutter in 9 cases (including 1 case converted to atypical atrial flutter and quickly restored sinus rhythm), with a restoration success rate of 81.8% (9/11) (see Figures 3, 4). One case of AFL presented with intermittent short paroxysms after TEAP restoration, and one case had an AV conduction ratio decreased to 3∼4:1, both continued oral treatment with digoxin combined with rhythm control medication. The median follow-up time of all neonates receiving antiarrhythmic drugs was 1.83 months (interquartile range: 0.83–2.25 months). All neonates successfully converted by TEAP remained arrhythmia-free throughout the follow-up period, with no recurrence of supraventricular tachycardia documented.

No TEAP-related complications occurred in this study, such as nasal injury, esophageal perforation, lung perforation, or intracranial misplacement of the electrode.

## Discussion

The management of continuous PSVT primarily involves the use of intravenous adenosine triphosphate disodium (4). In preterm infants especially, due to immaturity, cardiac arrhythmias can lead to hemodynamic decompensation within a short time (6). Treatment for SVT includes vagal maneuvers, antiarrhythmic drugs, TEAP, synchronized direct current cardioversion, and radiofrequency ablation (7). Currently, there are limited safe and effective antiarrhythmic drugs available for newborns. Continuous PSVT primarily uses intravenous adenosine triphosphate disodium (4). Propafenone is a commonly used class I antiarrhythmic drug for newborns, suitable for those with short-duration tachycardia without complications such as cardiac enlargement, heart failure, and hypotension (8). Despite its notable efficacy, the relatively high incidence of systemic adverse effects associated with amiodarone leads most pediatric cardiologists to reserve it for cases where SVT is refractory to other antiarrhythmic drugs. However, some authors advocate for its use as a first-line agent, particularly during infancy (9). For acute treatment failure, TEAP or even electrical cardioversion can be chosen to quickly restore sinus rhythm and maintain hemodynamic stability. TEAP has been mentioned as a temporary treatment option in refractory arrhythmias (10). In this study, the small sample size is primarily due to the very low incidence of refractory SVT in the neonatal population, combined with strict exclusion criteria that limited enrollment to cases where standard medical therapy had failed to achieve timely control. In our center, TEAP is employed as a rescue technique only after pharmacological failure, and it is not routinely utilized as a first-line therapy, which further contributed to the limited number of cases over the 10-year period. Eleven newborns diagnosed with PSVT via surface electrocardiogram, who failed to achieve rhythm restoration after vagal maneuvers and one or more antiarrhythmic drugs, were subsequently treated with TEAP.

Nasal insertion offers greater stability; however, for newborns weighing <3 kg, the nasal cavity is too small to accommodate a 7F catheter, necessitating oral insertion instead. After inserting the esophageal electrode catheter, 9 cases were diagnosed with AVRT and 2 with AT via transesophageal electrocardiography. Newborns with SVT typically have fast ventricular rates, with P waves buried in QRS complexes or T waves, making them difficult to identify on surface electrocardiograms. In contrast, P wave morphology on transesophageal electrocardiography is distinct and has high amplitude, making it easy to distinguish. TEAP and esophageal lead ECG can effectively identify, induce, and terminate supraventricular tachycardia by determining the relationship between P waves and QRS complexes, and synchronously measuring the RP interval and entrainment (2, 3, 11).

All 9 newborns with PSVT had AVRT, and there were no AVNRT cases in this study. After treatment, 1 case was confirmed to have manifest pre-excitation, while the other 8 had concealed bypass tracts. PSVT in newborns is often caused by manifest or concealed atrioventricular bypass tracts that lead to atrioventricular reentrant tachycardia (4, 12). In the two cases originally diagnosed with PSVT via surface electrocardiogram, P waves were clearly visible on transesophageal electrocardiography, and esophageal leads showed P'R < RP' and P'R < 1/2RR, thus confirming AT.

Transesophageal electrophysiologic studies are a highly accurate method for diagnosing and characterizing supraventricular tachycardia with various mechanisms in pediatric patients (13). The preliminary diagnostic results of transesophageal electrophysiology for tachycardia mechanisms are highly consistent with those of intracardiac electrophysiology (3, 13–15).

The mechanism of TEAP for PSVT is to deliver stimulation pulses into the excitable gap of the reentry circuit, form a new refractory period, and thereby block the antegrade or retrograde conduction of the reentry circuit to terminate tachycardia (16).

In this study, among the 9 AVRT patients, 5 had heart failure. TEAP successfully restored sinus rhythm in 7 cases, with a success rate of 77.8% (7/9). This rate is lower than the reported 88% success rate (15/17) for restoring sinus rhythm in newborns with PSVT (17), which may be attributed to the relatively small sample size of this group and the inclusion of patients with heart failure and severe conditions, leading to the difference in success rate.

TEAP for PSVT works rapidly, has no age restrictions, and does not suppress myocardial contractility. It is an emergency treatment option for patients with refractory SVT complicated with severe heart failure before extracorporeal membrane oxygenation is initiated (18). In this study, 1 case experienced recurrent persistent tachycardia after being terminated by esophageal pacing, so esophageal electrode catheters were retained. After multiple pacing sessions within 24 h, sinus rhythm was completely restored. TEAP can be performed at the bedside in emergencies and repeated as needed.

Two newborns with AVRT did not respond to TEAP treatment. One of them had complex congenital heart disease with heart failure, and the fastest ventricular rate reached 285 beats/min. After inserting the esophageal electrode catheter, an esophageal electrocardiogram was recorded for typing diagnosis. Pulse stimulation had not yet been administered, a declining trend in blood pressure was observed. Sinus rhythm was restored after 3 rounds of electrical cardioversion. Sustained tachycardia exacerbates cardiac insufficiency. One AVRT case had intermittent short paroxysmal episodes after sinus rhythm restoration, so oral medication was continued.

TEAP is feasible for children of all ages with atrial reentrant tachycardia, and short-duration atrial stimulation is highly effective for rapidly terminating AT (19, 20). However, in this study, TEAP failed to restore sinus rhythm in 2 newborns with AT. Analysis of the tachycardia formation mechanism suggests that the arrhythmia may be caused by increased automaticity rather than reentry. Subsequent dynamic electrocardiography recorded “gradual onset, gradual termination” during atrial tachycardia episodes in these two patients, confirming that TEAP is ineffective for tachycardia caused by increased automaticity.

AFL accounts for 30% of fetal rapid arrhythmias. AFL (often with 2:1 A-V conduction) may be difficult to detect on surface ECG, as P waves usually do not present a typical flutter morphology or are hidden within the T wave. Recordings via esophageal lead can clearly identify the underlying rhythm (19).

Drug therapy for atrial flutter has a slow onset and a long recovery time. Currently, there is no consensus or relevant evidence to support the optimal drug treatment regimen or treatment duration, and clinical trials focusing on drug therapy for this condition remain limited (21, 22). For hemodynamically unstable newborns, electrical cardioversion or transesophageal atrial pacing is the recommended intervention (23).

The occurrence of atrial flutter in newborns may be related to immature development of the cardiac conduction system, which leads to uneven atrial muscle refractory periods. Even a single premature stimulation can trigger reentry of electrical activation within the atrium. AFL circles clockwise or counterclockwise within the right atrium, with the cavo-tricuspid isthmus serving as the critical structural pathway (24). TEAP terminates AFL by interrupting the reentry circuit, and there are three possible responses after atrial flutter termination (25): (1) Atrial flutter directly converts to sinus rhythm; (2) Atrial flutter terminates after first converting to atrial fibrillation, then spontaneously restores sinus rhythm; (3) Atrial flutter converts to atrial fibrillation with a slow ventricular rate.

In this study, among 11 newborns with atrial flutter, 8 had a history of fetal tachycardia or suspected fetal distress. TEAP successfully restored sinus rhythm in 9 cases, with a success rate of 81.8%. Scholars recommend TEAP as the first-line method for acute treatment of atrial flutter in newborns, infants, and children due to its high success rate (19). External DC cardioversion should only be used to terminate atrial reentrant tachycardia when the esophageal approach fails, or when the patient is critically ill and requires immediate intervention (19). Two newborns had failed TEAP treatment and received continuous oral antiarrhythmic medication to control their ventricular rate.

Ajisaka et al. found that the success of TEAP in converting AFL is not significantly associated with atrial flutter duration, patient age, F wave amplitude, presence of primary heart disease, or heart failure. Instead, it is correlated with atrial flutter frequency: the slower the atrial frequency, the more likely AFL is to be terminated by atrial overdrive stimulation (26).

Complications of transesophageal recording and pacing are rare, and no mortality or long-term morbidity has been reported with the use of standard techniques (3, 27). Heart rate control via transesophageal atrial overdrive pacing is safe and effective, and helps infants with supraventricular tachycardia complicated by severe ventricular dysfunction restore normal hemodynamic status. This method can achieve immediate heart rate control, buy time for myocardial recovery, and enable safe implementation of antiarrhythmic drug treatment (15, 18).

This study shows that TEAP has achieved favorable clinical outcomes in the diagnosis and ablation of tachyarrhythmias in newborns. It avoids exposure to fluoroscopy and potential complications associated with transvenous catheterization, general anesthesia, and direct-current shock (26). TEAP is a relatively safe and effective acute treatment for reentrant SVT (AVRT, AFL) when the medication cannot promptly and effectively terminate the tachycardia, particularly for patients with heart failure.

## Data Availability

The original contributions presented in the study are included in the article/Supplementary Material, further inquiries can be directed to the corresponding authors.
